# OpenTox predictive toxicology framework: toxicological ontology and semantic media wiki-based OpenToxipedia

**DOI:** 10.1186/2041-1480-3-S1-S7

**Published:** 2012-04-24

**Authors:** Olga Tcheremenskaia, Romualdo Benigni, Ivelina Nikolova, Nina Jeliazkova, Sylvia E Escher, Monika Batke, Thomas Baier, Vladimir Poroikov, Alexey Lagunin, Micha Rautenberg, Barry Hardy

**Affiliations:** 1 Istituto Superiore di Sanità, Environment and Health Department, Viale Regina Elena 299, Rome 00161, Italy; 2 Ideaconsult Ltd, A. Kanchev 4, Sofia 1000, Bulgaria; 3 Fraunhofer Institute for Toxicology & Experimental Medicine, Nikolai-Fuchs-Str. 1, 30625 Hannover, Germany; 4 Orekhovich Institute of Biomedical Chemistry of Russian Academy of Medical Sciences, Pogodinskaya Street 10, 119121 Moscow, Russia; 5 In silico Toxicology, Altkircher Str. 4, CH-4052 Basel, Switzerland; 6 Douglas Connect, Baermeggenweg 14, CH-4314 Zeiningen, Switzerland

## Abstract

**Background:**

The OpenTox Framework, developed by the partners in the OpenTox project (http://www.opentox.org), aims at providing a unified access to toxicity data, predictive models and validation procedures. Interoperability of resources is achieved using a common information model, based on the OpenTox ontologies, describing predictive algorithms, models and toxicity data. As toxicological data may come from different, heterogeneous sources, a deployed ontology, unifying the terminology and the resources, is critical for the rational and reliable organization of the data, and its automatic processing.

**Results:**

The following related ontologies have been developed for OpenTox: a) Toxicological ontology – listing the toxicological endpoints; b) Organs system and Effects ontology – addressing organs, targets/examinations and effects observed in *in vivo* studies; c) ToxML ontology – representing semi-automatic conversion of the ToxML schema; d) OpenTox ontology– representation of OpenTox framework components: chemical compounds, datasets, types of algorithms, models and validation web services; e) ToxLink–ToxCast assays ontology and f) OpenToxipedia community knowledge resource on toxicology terminology.

OpenTox components are made available through standardized REST web services, where every compound, data set, and predictive method has a unique resolvable address (URI), used to retrieve its Resource Description Framework (RDF) representation, or to initiate the associated calculations and generate new RDF-based resources.

The services support the integration of toxicity and chemical data from various sources, the generation and validation of computer models for toxic effects, seamless integration of new algorithms and scientifically sound validation routines and provide a flexible framework, which allows building arbitrary number of applications, tailored to solving different problems by end users (e.g. toxicologists).

**Availability:**

The OpenTox toxicological ontology projects may be accessed via the OpenTox ontology development page http://www.opentox.org/dev/ontology; the OpenTox ontology is available as OWL at http://opentox.org/api/1 1/opentox.owl, the ToxML - OWL conversion utility is an open source resource available at http://ambit.svn.sourceforge.net/viewvc/ambit/branches/toxml-utils/

## Background

### Introduction

The field of predictive toxicology urgently requires the development of open, public, computable standardised toxicology vocabularies and ontologies to support the applications required by *in silico*, *in vitro* and *in vivo* toxicology methods and related reporting activities such as the REACH (Registration Evaluation and Authorisation of Chemicals) legislation [1]. One important goal of OpenTox is to meet the requirements of the REACH legislation using alternative testing methods to contribute to the reduction of animal experiments for toxicity testing.

All predictive approaches in toxicology share the need of highly-structured information as a starting point. The definition of ontology and of controlled vocabulary is a crucial requirement in order to standardize and organize the chemical and toxicological databases on which the predictive toxicology methods build on, to improve the interoperability between toxicology resources and to create a knowledge infrastructure supporting R&D and risk assessment.

OpenTox (OT) [2] was funded by the EU Seventh Framework Program (FP7) to develop a framework for predictive toxicology modelling and application development. The framework consists of distributed web services, running at several locations. Two initial OT web-applications have been made available, based on this framework: ToxPredict [3] that predicts the activity of a chemical structure submitted by the user in respect to a given toxicity endpoint, and ToxCreate [4] that creates predictive toxicology models from a user-submitted dataset. Bioclipse, an Open Source workbench for the life sciences, was extended to launch calculations on remote OT services and to provide a rich user interface on the desktop [5].

Initial analysis on OpenTox highlighted the importance of the standardisation of the framework components for describing both toxicity data and computational procedures. Ontology definition is important for OT, as it supports integrated information processing in a more efficient and reliable manner, thus reducing the cost, maintenance and risk of application development and deployment.

### Related projects

Numerous existing ontologies and standard initiatives can contribute to the creation of a toxicology ontology supporting the needs of predictive toxicology and risk assessment. We briefly review a number of relevant related projects here.

Computational tools for predictive toxicology include a range of well-known machine learning and bioinformatics algorithms, as well as specific cheminformatics procedures, such as for descriptor calculation and chemical structure processing. The Blue Obelisk descriptor ontology [6] was the first attempt to provide a formal description of some cheminformatics algorithms. It was adopted in OpenTox, and was further extended, in order to incorporate algorithms not available in the original version. The Chemical Information Ontology is another ontology, which was published [7], and is considered the successor of the Blue Obelisk descriptor ontology. However, it is not yet used in OpenTox, as it only became available recently. Similarly, the lack of ontologies, covering machine learning and data mining domains at the beginning of the project, led to the independent development of the OpenTox ontology [2], representing the core components of the OpenTox framework, as datasets, features, tasks, algorithms, models and validation. We were not aware at that time of DAMON [8], developed in the context of Grid services and available in DAML+OIL instead of OWL that makes this ontology harder to reuse. Despite having been built in the context of predictive toxicology, the OpenTox ontology shares several similarities with published data mining ontologies - the ontology of data mining (OntoDM) ontology [9,10], KDDOnto [11], KDO ontology [12], DMWF Ontology [13], and the e-LICO Data Mining Ontology (DMO), developed in the framework of another EU FP7 project [14]. OntoDM is based on the unification of the field of data mining and the growing demand for formalized representation of outcomes of research. It includes definitions of basic data mining entities, such as datatype, dataset, data mining task, data mining algorithm and components thereof (e.g., distance function), etc. OntoDM also allows the definition of more complex entities, e.g., constraints in constraint-based data mining, sets of such constraints (inductive queries) and data mining scenarios. The e-LICO team launched the Data Mining Ontology Foundry [15], which is populated with e-LICO suite of ontologies for data mining (DMO), model selection and meta-mining (Data Mining Optimization – DMOP) [16]. DMO also includes similar basic data mining entities, and provides means to automatically compose workflows by identifying algorithms with compatible input and output. Finally, collecting details of machine learning experiments in “experiment databases” for subsequent analysis [17,18], is comparable to the OpenTox framework design, which provides distributed storage for all details of predictive toxicology workflows.

Despite the surge of simultaneous activities in developing data mining ontologies, their adoption by the major data mining platforms and tools is still a future goal.

Unifying toxicology data description presents additional challenges.

As one of the central repositories of large-scale biomedical ontologies, the OBO Foundry [19] is an important source of ontologies for reuse. Several OBO ontologies could potentially be used as part of the development of a Toxicology Ontology.

The Gene Ontology (GO) [20] project is a collaborative effort to address the need for consistent descriptions of gene products in different databases. The GO project has developed three structured controlled vocabularies that describe gene products in terms of their associated biological processes, cellular components and molecular functions in a species-independent manner.

Chemical Entities of Biological Interest (ChEBI) [21] is a freely available dictionary of molecular entities focused on “biologically interesting” chemical entities and their activities in a biological context. The molecular entities in question are either natural or synthetic products used to intervene in the processes of living organisms.

The Ontology of Biomedical Investigations (OBI) [22] provides terminology relevant to experimental biological and clinical investigations. This includes a set of 'universal' terms, applicable across various biological and technological domains, and domain-specific terms. This ontology supports the consistent annotation of biomedical investigations, regardless of the particular field of study. The OBI addresses the need for a cross-disciplinary approach and represents all phases of experimental processes, and the entities involved in preparing for, executing, and interpreting those processes e.g., study designs, protocols, instrumentation, biological material, collected data and analyses performed on that data.

Other existing ontologies of relevance to the toxicology domain include anatomy ontologies such as the Foundational Model of Anatomy (FMA) [23] and the Mouse adult gross anatomy ontology [24].The FMA Ontology is a knowledge source for biomedical informatics; it is concerned with the representation of classes or types and relationships necessary for the symbolic representation of the phenotypic structure of the human anatomy. Its ontological framework can be applied and extended to all other species. The Mouse adult gross anatomy ontology represents the Anatomical Dictionary for the Adult Mouse. This ontology organizes anatomical structures for the postnatal mouse spatially and functionally, using 'is a' and 'part of' relationships. A browser can be used to view anatomical terms and their relationships in a hierarchical display.

One more toxicology-relevant ontology is the NCI Thesaurus [25], which contains terminology relevant for clinical care, translational and basic research, and public information and administrative activities, with respect to cancer and related diseases and targeted therapies. The NCI Thesaurus provides definitions, synonyms, and other information on nearly 10,000 cancers and related diseases, 8,000 single agents and combination therapies, and a wide range of other topics related to cancer and biomedical research.

The National Center for Biomedical Ontology (NCBO) hosts the BioPortal that is another important ontology repository. The BioPortal provides access to ontologies of interest to the biological and biomedical community including the large-scale terminology standards such as MeSH [26] and SNOMED [27]. MeSH (Medical Subject Headings) is the controlled vocabulary thesaurus used for indexing articles for PubMed SNOMED CT (Systematized Nomenclature of Medicine -- Clinical Terms), which is a systematically organised computer-processable collection of medical terminology covering most areas of clinical information such as diseases, findings, procedures, microorganisms, and substances. It allows a consistent way to index, store, retrieve, and aggregate clinical data across specialties and sites of care. It also helps organising the content of medical records, reducing the variability in the way data is captured, encoded and used for clinical care of patients and research.

The eTOX project [28] aims to develop a drug safety database from pharmaceutical industry legacy toxicology reports and public toxicology data. Ontology development within the eTOX Project includes the activity to create ontologies for preclinical safety.

There are two important toxicological standards that are publically available: the OECD harmonized templates (OECD HTs) [29] and the ToxML (Toxicology XML standard) schema (initiated by Leadscope Inc.) [30].

The OECD HTs correspond to the IUCLID5 XML [31] schemas, which are meant to be used by industry when submitting documentation on their chemicals to EU regulatory authorities. For each endpoint, the OECD HTs define a series of fields e.g., defining the information submission requirements of a carcinogenicity experiment. Since they are generic enough to be able to include data on endpoints with different characteristics, in principle the OECD HTs provide a substantial basis for building ontology. However, they are not very formalized; they leave much space for free text entering, and have a strong administration emphasis rather than a scientific focus.

ToxML is an XML data exchange standard based on toxicity controlled vocabulary. The most recent ToxML release has a comprehensive, well-structured scheme for many toxicity studies (carcinogenicity, *in vitro* mutagenicity, *in vivo* micronucleus mutagenicity, repeated dose toxicity) which fit well the OpenTox purposes. For this reason we decided to explore the possibility of semi-automatic conversion of the ToxML schema to OWL-DL within OT, with the purpose of benefitting from the reasoning mechanism of OWL. The resulting ontology will be applied to reference and annotate the contents of databases coming from various sources and toxicity studies.

Even though all the ontologies described above exist, there is no systematic ontology for toxicological effects and predictive toxicology needs. The aim of the OpenTox ontology is to standardize and organize chemical and toxicological databases and to improve the interoperability between toxicology resources processing this data.

Even if several ontologies covering the anatomy domain exist, there is a serious gap for localized histopathology, and more generally, ontologies of micro anatomy. For this reason the Organs and Effects ontology has been developed within OpenTox. This ontology is closely linked to the INHAND initiative (International Harmonization of Nomenclature and Diagnostic Criteria for Lesions in Rats and Mice). INHAND aims to develop for the first time an internationally accepted standardized vocabulary for neoplastic and non-neoplastic lesions as well as the definition of their diagnostic features. The description of the respiratory system [32] is already implemented in the OT ontology; additionally, the terms and diagnostic features of the hepatobiliary system were published [33].

### OpenTox and ontology need

A predictive toxicology framework essentially needs to provide modelling and predictive capabilities, and data access to chemical structures and toxicity data. From an ontology development point of view, data mining ontologies are relevant for the former, and ontologies, handling representation of chemical entities and biological data, are required for the latter. The data mining ontologies are usually developed from an abstract point of view; since the relevant algorithms and data structures are independent of the specific domain they could be applied to. While this approach certainly has its advantages, the ultimate result of only using data mining concepts to represent a predictive toxicology model is that the biological context is stripped off.

A predictive toxicology model, reporting whether a chemical compound is carcinogenic or not, would be represented as a classification one, trained by a given classification algorithm and predicting a binary outcome. The training dataset would be represented most often in a matrix form, with the provenance information related to how the carcinogenicity measurements had been taken either discarded, or in the best case, described in a human readable form in the accompanying documentation only. This is sufficient to build the model and assess its performance, but is less useful for the end users, who are experts in toxicology, but not in modelling algorithms.

On the other hand, toxicology studies are represented in much more detail in specialized databases, but data exchange formats rarely make use of structured formats or ontological representation. Moreover, they are not directly useful for processing by data mining software tools.

We defined the OpenTox ontology to represent datasets and properties of chemicals by unified means, suitable for the modelling algorithms.

To summarize, the ontology development in the OpenTox framework is not an end goal by itself, but an inherent part of retaining the biological context in machine learning datasets and keeping track of the data provenance, as it is passed through various processing methods.

The OpenTox ontology aims to cover from a semantic point of view the toxicological endpoints and experimental databases included in the OT final database. The data sources have been selected within publicly available data sources, providing high-quality structural and/or toxicological data. There are currently no standard datasets in this area and for this reason the purpose of the OT ontology was to integrate all these heterogeneous databases together. One of the important datasets considered for the construction of the various ontologies was the DSSTox CPDBAS (Carcinogenic Potency Database) [34]. Another example of such a data source is the ISSCAN database [35] developed by the OT partner Istituto Superiore di Sanità (ISS). This database originates from the experience of researchers in the field of structure-activity relationships (SAR), aimed at developing models which theoretically predict the carcinogenicity of chemicals.

These two public and widely known datasets mentioned above show the typical scenario of the current state of representing toxicity data. Both datasets are available as SDF files, with fields described in human readable documents only. The outcome of the carcinogenicity study is represented in the "ActivityOutcome" field in CPDBAS (with allowed values "active", "unspecified", "inactive"), while in ISSCAN, a numeric field named "Canc" is used with allowed value of 1, 2, or 3. The description of the numbers (3 = carcinogen; 2 = equivocal; 1 = non-carcinogen) is only available in a separate "Guidance for Use" pdf file. Ideally, toxicity prediction software should offer comparison between the data and models, derived from both datasets, which is impossible without involving human efforts to read the guides and establish the semantic correspondence between the relevant data entries if and when possible.

### OpenToxipedia

OpenToxipedia [36] is a new community resource of toxicology terminology organized by means of a Semantic Media Wiki (SMW). OpenToxipedia supports creating, adding, editing and maintaining terms used in both experimental toxicology and *in silico* toxicology. The particular importance of OpenToxipedia relies on the description of all the terms used in OT applications such as ToxPredict and ToxCreate.

## Methodology

The construction of formal ontology follows relatively established principles in knowledge representation. We have taken into consideration those principles available for biomedical ontology development, particularly the OBO Foundry principles, such as availability for community, common syntax, collaborative development, documentation and definition of terms. We have tried to keep our ontology orthogonal to other existing projects. OT ontologies were checked for logical consistency with the Pellet OWL reasoned [37]. Our open approach to ontology development supports current and future collaborations with different projects. We use the DL species of the Web Ontology Language (OWL DL) supported by the Protégé OWL editor. An overview of the OT ontology is given on the public area of the OT website [38] together with instructions on how to enter the OT Collaborative Protégé Server and to contribute to existing OT projects on OWL development. Some of the ontologies are manually created from scratch; others partially reuse existing ones and extend them with task-related concepts and relations.

The ToxML ontology is semi-automatically generated from the existing ToxML schema by parsing it to OWL. The sub-schemas describing different toxicity studies were analysed by chemists and computer scientists who agreed on a set of rules which needed to be implemented to convey the semantics of the relations between the objects and to remove redundant information in the new format. The rules are directions for creating, removing, and renaming classes/properties which are to be executed by the program and they cover various aspects such as:

• to distinguish classes from properties among the XML fields;

• to introduce object properties – by default in the schema all properties correspond to data type properties in OWL because they connect an entity to a string value;

• to remove some of the container classes, which are not needed in an ontology (Tests, Compounds, etc.) – these are necessary in XML because they frame a set of subfields, but in OWL, each *Test* or *Compound* is a separate Object and many of these objects can exist independently and they are all related to their originating type class;

• to rename classes which appear with the same name in different contexts.

**Figure 1:**
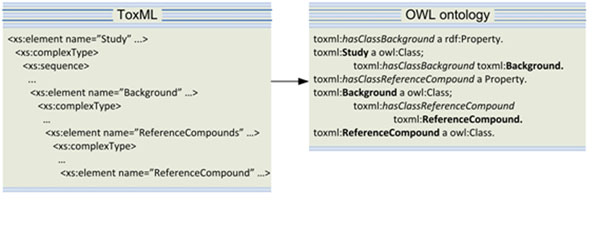
Introducing object type properties for each tuple of nested classes

The resulting ontology has a flat structure with numerous newly introduced relations (of type rdf:Property) representing the semantics of the nested structure of the XML ToxML schema (see Figure 1);

• The IS-A relation is introduced only to a limited number of classes.

**Example: ChronicStudies ***rdfs:subClassOf ***Study**;

• The relations between the classes are obtained from the nested XML structures and encoded as follows: for each tuple of nested fields in the schema *F*_1_ and *F*_2_ (nested in *F*_1_), two new classes <Class *F*_1_> and <Class *F*_2_> are created in OWL along with an object property <*hasClassF_2_*> which expresses the relation between both classes. The feature <*hasClassF_2_*> has domain <Class *F*_1_> and range <Class *F*_2_> (the range and domain could be a union of classes if the nested class appears more than once in the source schema). For example Figure 1 compares the corresponding representation of the nested fields Study, Background and ReferenceCompound in ToxML and OWL.

• Fields which have string values in XML are converted to properties in OWL.

**Example:** The field *Name* which has a string value type becomes a datatype property *hasName*

• Ambiguous labels are unified.

**Example:** The field *Results* is used in several different contexts, that is why we rename it to *TreatmentResults*, in the treatment group;

• Wherever possible, object type properties are introduced instead of datatype ones – thus string values are replaced by named concepts.

**Example:** In ToxML schema the field *Sex* is defined as a simple type of type string, which would be converted to property in direct conversion, but in OWL we introduce a new class Sex, thus creating an object property *has Sex* instead of a datatype one.

• Mapping to Organ ontology and study type classifications can be applied.

OpenToxipedia has been developed using the SMW. It was created manually by experts in the fields of *in silico* and experimental toxicology on the basis of known regulatory documents, glossaries, dictionaries and some primary publications. All registered members are welcome to add new entries, suggest definitions and edit the existing resource at http://www.opentoxipedia.org. OpenToxipedia is curated by toxicology experts within the OT community.

The SMW was chosen for OpenToxipedia representation for the following main reasons: it enables automatic processing of the wiki knowledgebase and it gives a possibility for data transfer between RDF and SMW through SPARQL. SMW will facilitate the automatic data exchange between OpenToxipedia, the ontologies and OpenTox web services using RDF data. The SMW is a collaborative system, supports versioning, RDF export, tools to lock pages by a curator (fixing a validated vocabulary) and the possibility to add annotation without changing the ontology or RDF information.

## Results and discussion

### Sub-ontology projects developed within the OpenTox project

Up to now, six ontologies have been made available through the OT Collaborative Protégé Server:

• Toxicological Endpoint ontology;

• Organs system and Effects ontology;

• ToxML ontology;

• OpenTox ontology, representing components of OpenTox web services, framework and Algorithm types;

• ToxLink (ToxCast [39] assays ontology);

• OpenToxipedia: SMW toxicology knowledge resource.

### Toxicological Endpoint ontology

**Figure 2:**
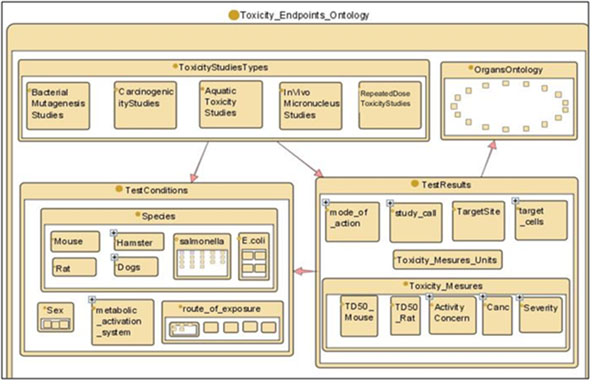
OpenTox toxicological ontology structure

The OT Toxicological Endpoint ontology contains five toxicity study types: carcinogenicity, *in vitro* bacterial mutagenicity, *in vivo* micronucleus mutagenicity, repeated dose toxicity (e.g., chronic, sub-chronic or sub-acute study types) and aquatic toxicity (see Figure 2).

The purpose of this ontology is to enable the attributes of toxicological dataset entries to be associated with ontology concepts. The OT framework exposes REST web services, corresponding to common OT components. A generic OWL representation is defined for every component (e.g. every OT dataset is a subclass of ot:Dataset, every algorithm is subclass of ot:Algorithm and every model is a subclass of ot:Model). This allows unified representation across diverse data and algorithms, and a uniform interface to data processing services, which take generic ot:Dataset resources on input and generate generic ot:Dataset resources on output.

The main OWL classes are “ToxicityStudyType”, “TestSystem” (includes subclasses such as strains, species, sex, route of exposure), “TestResult” (includes subclasses such as toxicity measure, test call, mode of action, target sites).

### Organs and Effects ontology

The Organs system ontology was developed by the Fraunhofer Institute for Toxicology & Experimental Medicine (ITEM), and will be linked with the above-mentioned “target sites” toxicological class (work-in-progress). The Organs system ontology is one of the most challenging ontology classes addressing targets, organs and effects observed in *in vivo* studies such as repeated dose toxicity and carcinogenicity experiments. The ontology includes the detailed description of organs starting from organs systems down to histological components. It was decided to use a hierarchical structure starting with the organs system (e.g. digestive system) instead of orientating the ontology on the examinations performed in guideline studies such as histopathology, necropsy, and clinical observations. The basic structure of the organs ontology is as follows:

- class organs system - subclass organs system

|- class anatomical structure - subclass anatomical structure 1 to n

|- class of synonyms cross-linked to terminology

|- class of main cell types in organ tissue of resp. and dig. tract (to be extended)

|- class of main hormones and enzymes of resp. and dig. tract (to be extended)

The Organs system ontology includes 13 organ systems: digestive system, respiratory system, circulatory system, endocrine system, male genital system, female genital system, hematopoietic system, integumentary system, body cavities, nervous system and special sense organs, urinary system, musculoskeletal system, immune system and lymphatic organs. Synonyms are included to account for differences in terminologies. It focuses on the organs observed in rodents, which are frequently used for toxicity testing. Species specificity will be introduced, when the Organs systems ontology is combined with the Toxicological Endpoint ontology.

**Figure 3:**
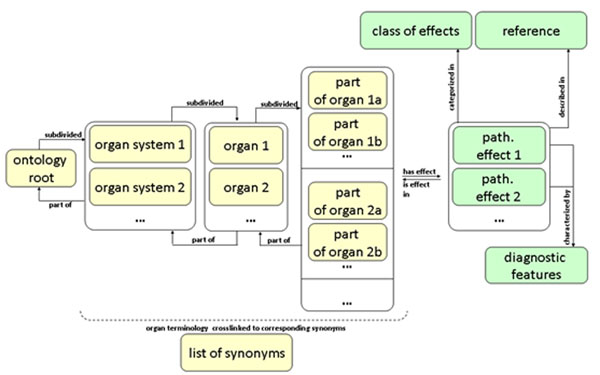
Overview of the structure of the combined Organs system (in orange) and Toxicological Effects (in green) ontologies

The Toxicological Effects Ontology comprises neoplastic and non-neoplastic effects observed in repeated dose and cancer studies. This ontology consists of three main parts: classes of effects, linked to pathological effects, which are further linked to detailed diagnostic features as agreed in the INHAND initiative. Its functionality has been initially developed for the respiratory tract. The structure of the combined Organs system and Effects ontology is depicted in Figure 3.

### ToxMLOntology

The ToxML ontology is a semi-automatic conversion of ToxML schema to OWL-DL. The most recent ToxML release has a comprehensive, well-structured scheme for many toxicity studies (carcinogenicity, *in vitro* mutagenicity, *in vivo* micronucleus mutagenicity, repeated dose toxicity) which fit well the OpenTox purposes. This was verified by manually mapping various existing database entries to the ToxML schema. Our purpose is not only to develop a cross database matching schema but also to inference on existing facts in the databases.

In order to use ToxML for data annotation we needed to overcome issues raised by the nature of the XML description. In the schema many fields host free text, instead of named concepts which hampers further automatic processing.

***Example:*** In carcinogenicity studies data the survival rate is described as “*MORTALITY*, *INCREASED*” that are plain text strings not referring to any conceptual knowledge.

For solving this issue, in the ontology the same values are assigned as properties of a concept which is located within a domain knowledge model, thus allowing referencing between known entities. Another problem was the mixed type values (string / integer / interval) describing the same indicator in different studies.

***Example:*** In data from different chronic studies the same indicator survival rate is presented as either of the following strings:

'0/5 (weeks 27-30)' ;'10/10' ; '16/17' ; '5/5' ; '6/6'

The first string in the example '0/5 (weeks 27-30)' represents the number of survived animals among the tested ones during some concrete period of the study between the 27^th^ and 30^th^ week, whereas in all other cases the strings show only the number of survived animals in relation to all tested animals. These are inconsistent data records which show the same indicator value in different value types. Organizing the type values as restricted values in the ontology allows for comparison and further automatic processing which is not feasible in plain string.

The vocabulary used for labelling the classes will be further standardized by partially applying already existing controlled terminologies for target sites, mode of action, and route of exposure. In XML schema sometimes the same field names are used to label classes and properties. Introducing controlled vocabularies helps avoiding term overlapping and ambiguity.

**Figure 4:**
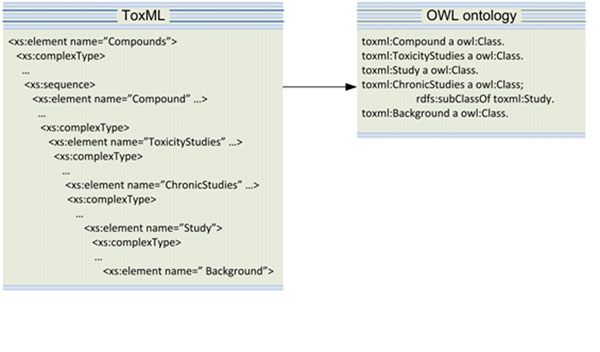
Nested structures in OWL converted to flat structure without sub-classing in OWL

The classes in the OWL ontology are interconnected by newly created object properties (as explained in the Methodologies section) and rarely by the IS-A relation because the XML nested structure does not follow the natural IS-A relation used for sub-classing in OWL. Figure 4 shows the differences between the XML and OWL representation of the same facts.

### OpenTox Ontology: the OpenTox web services framework and algorithm types ontology

The OpenTox ontology [40] provides a common information model for the most common components, found in any OT application, providing predictive toxicology functionality, namely chemical compounds, datasets of chemical compounds, data processing algorithms, machine learning algorithms, predictive models and validation routines. It is available as OWL at http://opentox.org/api/1 1/opentox.owl and described in detail in [1]. The OpenTox framework exposes REST web services, corresponding to each of these common components. A generic OWL representation allows unified representation across diverse data and algorithms, and a uniform interface to data processing services, which take generic ot:Dataset resources on input and generates generic ot:Dataset resources on output. Specific types of algorithms are described in the algorithm types ontology and more details of descriptor calculation algorithms are specified via the Blue Obelisk ontology [6] of cheminformatics algorithms (e.g. algorithm references, descriptor categories) and extensions, specifically developed to cover algorithms developed by OpenTox developers. Assigning specific information about the datasets, properties and types of algorithms and models is done via linking to the relevant ontologies, for example by sub-classing (rdf:type), owl:sameAs links, or Blue Obelisk ontology bo:instanceOf predicate.

The simultaneous use of OT datasets and compound properties as resources of generic ot:Dataset type and ot:Feature type in the OT ontology, and linking to specific toxicology ontologies, provides a flexible mechanism for annotation. It allows users of OT web services to upload datasets of chemical compounds and arbitrarily named properties of the compounds. The datasets are converted into a uniform ot:Dataset representation and chemical compound properties annotated with the proper terms from toxicology ontologies. The annotation and assigning of owl:sameAs links is only done manually, via OT REST web service interface, which modifies the relevant resource representation by adding/modifying triples. In principle, more sophisticated techniques could be applied, and the corresponding RDF representation updated via the same REST interface. This approach is used to enter and represent data in OT services and applications. Description of one of the OT API implementations, and examples of RDF representation of various resources is provided in paper [41].

The OpenTox ontology service provides a place for registering OpenTox resources, running at remote places, as well as searching capabilities via SPARQL. This allows a model, created by a remote service, to become available to any application that can send relevant queries to the ontology service. The registered resources and their properties could be retrieved via the service SPARQL endpoint. Adding query conditions may restrict the search to data or models of specific type (e.g. regression) or to the specific toxicology endpoint of interest.

**Figure 5:**
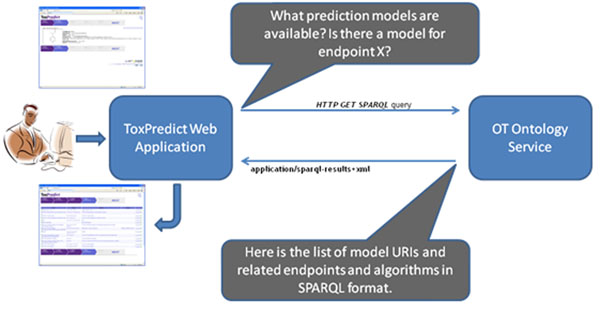
ToxPredict and Ontology Service (behind the scenes)

A typical workflow, as represented by a graphical user interface (such as ToxPredict or ToxCreate), includes querying an ontology service to find models or data of interest to the user, applying the processing via the OpenTox API (and eventually triggering a chain of processing by remote services) and finally presenting the prediction results, decorated by both data mining and biological context, as represented by respective ontologies (see Figure 5).

**Figure 6:**
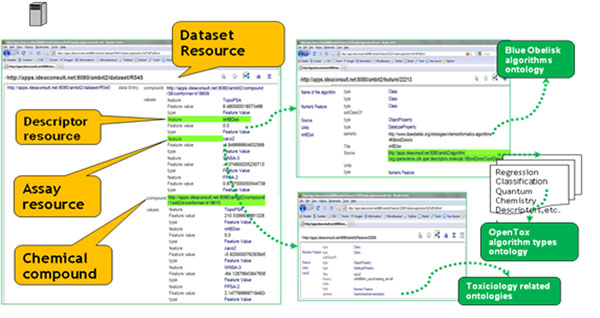
OpenTox linked resources and Ontology use: Compound, Algorithm, Model, Dataset, Features

A diagram explaining the OpenTox process and workflow involved in predicting toxicity using linked resources is shown in Figure 6.

The OpenTox framework provides not only the missing link between data mining and biological concepts in the case of predictive toxicology models, but also consists of a distributed set of communicating web resources, dynamically consuming and generating RDF triples, defined by the relevant ontologies. Anybody could independently bring online a new dataset, annotated with appropriate ontologies, a new machine learning algorithm or a predictive model, following the OpenTox API. The services, implementing the OpenTox API for algorithms and models, provide a unified web service interface to several descriptor calculations, machine learning and similarity searching algorithms, as well as to applicability domain estimation and diverse toxicity prediction models. Future predictive models may integrate reasoning capabilities, but still use the same uniform representation. The complexity and diversity of the processing is reduced to the simple paradigm "read data from a web address, perform processing, write to a web address". RDF representation of all resources can be retrieved via their web addresses and analysed automatically. The OpenTox components can work equally well across the internet, inside an intranet or on a single stand-alone machine. There is a striking parallel with the ideas of “down scaling” the semantic web, looking for the solutions of moving away from heavy and centralized triple stores, but still retaining the power of structured representations and semantic technologies. To summarize, the ontology development in OpenTox framework is not an end goal by itself, but an inherent part of retaining the biological context in machine learning datasets and keeping track of the data provenance, as it is passed through various processing methods.

### ToxCast *in vitro* toxicological assays ontology

The sixth ontology project initiated was the ToxLink ontology representing the ToxCast assays from the US EPA. This development is a collaborative effort of OpenTox with ToxCast [39] to provide an ontological description of *in vitro* toxicological assays.

### OpenToxipedia

**Figure 7:**
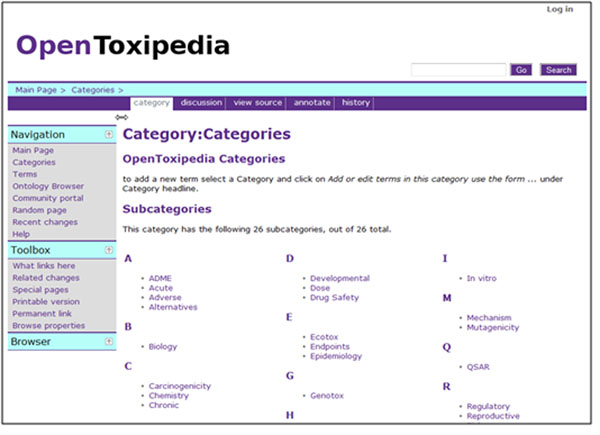
OpenToxipedia categories for predictive toxicology

**Figure 8:**
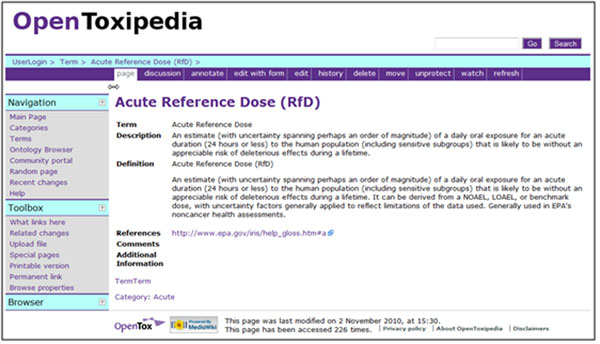
The interface for Open Toxipedia term management

OpenToxipedia is an important part of the OpenTox project that supports the definition of terms used in the developed ontologies, OpenTox web services and OpenTox applications such as ToxPredict and ToxCreate. At present, OpenToxipedia contains 862 toxicological terms with descriptions and literature references classified into 26 categories (see Figure 7). The terms can be browsed either by category or in alphabetical order. Due to its open collaboration platform, specialists in different toxicology fields can take part in the further creation and curation of terms in OpenToxipedia (see Figure 8).

OpenToxipedia provides a compendium for freely-available predictive toxicology resources supporting the application and development of the standards for representation of toxicology data, vocabulary and ontology development needed by OpenTox use cases and web services. The following rules for term management in OpenToxipedia have been developed: (*i*)Add terms – any registered user (curators receive a message and decide what additions are approved and will become publicly available); (*ii*) Edit description of terms – curators; (*iii*)Add remarks - any registered user (curators receive an alert message).

### Use case example

The RepDose [37] dataset implemented in Ambit for OpenTox [34] contains information on 583 chemicals. An instance of AMBIT services, implementing OpenTox API was installed at Fraunhofer ITEM server, and Repdose data imported via standard OpenTox API for uploading data and chemical structures. This makes the data available to OpenTox partners via the uniform OpenTox interface. The Repdose dataset is annotated by Organs and Effects Ontology. RDF/N3 annotation of some of the Repdose data is shown in Additional file 1.

## Conclusions

The need for speeding up the toxicological assessment of chemicals, and of using less animals and less expensive tools has strongly stimulated the development of predictive toxicology and of structure-based approaches. A wide spectrum of predictive approaches applied to toxicity exist today, including read-across, regulatory categories, and (Quantitative) Structure-Activity Relationships ((Q)SAR) modelling. All these predictive approaches share the need of highly structured information as a starting point: the definition of ontology and of controlled vocabulary is a crucial requirement in order to standardize and organize the chemical and toxicological data on which the predictive toxicology methods build on. The availability of ontology specific for predictive toxicology is crucial to the interoperability of OpenTox services and data resources. OT ontology could be integrated for other software platforms in developing and deploying user applications, thus laying the foundations for a semantic web linking effectively all the resources of the growing field of 21^st^ Century predictive toxicology.

The initial OpenTox FP7 project was completed in August 2011. We hope the OT ontology will be incorporated in an extended future toxicological ontology framework. There is an important need for an increased international coordination of efforts for the integration of genomics, proteomics, transcriptomics data that would provide the possibility of identifying the potential targets involved in pathological processes and of selecting the most promising targets for future chemical or drug product development.

Future predictive models may integrate reasoning capabilities, but still using the same uniform representation. The complexity and diversity of the processing is reduced to the simple paradigm "read data from a web address, perform processing, write to a web address". RDF representation of all resources can be retrieved via their web addresses and analysed automatically. The OpenTox components can work equally well across the internet, inside an intranet or collapsed into a local application running on a single machine. There is a striking parallel with the ideas of “down scaling” the semantic web looking for the solutions of moving away from heavy and centralized triple stores, but still retaining the power of structured representations and semantic technologies.

## Competing interests

The authors declare that they have no competing interests.

## Authors' contributions

BH supervised the Project, OLT and RB worked on the Toxicological Endpoint ontology, OLT coordinated the OpenTox ontology working group, NJ worked on the OpenTox ontology and on representation of the OpenTox framework components, SE, MB and TB worked on the Organs system and Effects Ontology, IN worked on the ToxML ontology and implemented a semi-automatic conversion of the ToxML schema, MR was responsible for administration of the Collaborative Protégé Server, VP and AL worked on the OpenToxipedia and on the Aquatic toxicity ontology that is included in the Toxicological Endpoint ontology. All authors revised and approved the final manuscript.

## Supplementary Material

Additional file 1 The Repdose database annotated by Organs and Effects Ontology in RDF/N3 format. Click here for file
