# Supplementary material for: OpenTox predictive toxicology framework: toxicological ontology and semantic media wiki-based OpenToxipedia
Source: J Biomed Semantics. 2012 Apr 24;3(Suppl 1):S7. doi: 10.1186/2041-1480-3-S1-S7 (PMC3337268; doi:10.1186/2041-1480-3-S1-S7)
Supplement: Additional file 1 — The Repdose database annotated by Organs and Effects Ontology in RDF/N3 format. [file 2041-1480-3-S1-S7-S1.docx]

**Additional file 1**

The Repdose database annotated by Organs and Effects Ontology in RDF/N3 format.

@prefix ot: <http://www.opentox.org/api/1.1#> .

@prefix dc: <http://purl.org/dc/elements/1.1/> .

@prefix : <https://ambit2.repdose-opentox.de:8443/ambit2/> .

@prefixota:<http://www.opentox.org/algorithmTypes.owl#> .

@prefix otee: <http://www.opentox.org/echaEndpoints.owl#> .

@prefix dcterms: <http://purl.org/dc/terms/> .

@prefix rdfs: <http://www.w3.org/2000/01/rdf-schema#> .

@prefix owl: <http://www.w3.org/2002/07/owl#> .

@prefix xsd: <http://www.w3.org/2001/XMLSchema#> .

@prefix rdf: <http://www.w3.org/1999/02/22-rdf-syntax-ns#> .

<https://ambit2.repdose-opentox.de:8443/ambit2/feature/58>

aot:Feature ;

dc:title "g_Organ" ;

ot:hasSource<https://ambit2.repdose-opentox.de:8443/ambit2/dataset/13> ;

ot:units "" ;

= <http://www.owl-ontologies.com/Ontology1312197749.owl#s03_organ_structure_level_I> .

<https://ambit2.repdose-opentox.de:8443/ambit2/feature/57>

aot:Feature , ot:NumericFeature ;

dc:title "f_Study LOEL in mmol" ;

ot:hasSource<https://ambit2.repdose-opentox.de:8443/ambit2/dataset/13> ;

ot:units "mmol" ;

= <http://www.opentox.org/Ontology.owl#LOEL> .

<https://ambit2.repdose-opentox.de:8443/ambit2/feature/49>

aot:Feature ;

dc:title "b_Study" ;

ot:hasSource<https://ambit2.repdose-opentox.de:8443/ambit2/dataset/13> ;

ot:units "" ;

= <http://www.opentox.org/Ontology.owl#ToxicityStudiesTypes> .

ot:Feature

aowl:Class .

<https://ambit2.repdose-opentox.de:8443/ambit2/feature/59>

aot:Feature ;

dc:title "d_Study Reliability" ;

ot:hasSource<https://ambit2.repdose-opentox.de:8443/ambit2/dataset/13> ;

ot:units "" ;

= <http://www.opentox.org/api/1.1#d_Study+Reliability> .

<https://ambit2.repdose-opentox.de:8443/ambit2/feature/50>

aot:Feature ;

dc:title "i_Effect Sex" ;

ot:hasSource<https://ambit2.repdose-opentox.de:8443/ambit2/dataset/13> ;

ot:units "" ;

= <http://www.opentox.org/Ontology.owl#Sex> .

<https://ambit2.repdose-opentox.de:8443/ambit2/feature/51>

aot:Feature , ot:NumericFeature ;

dc:title "j_Effect LOEL in mmol" ;

ot:hasSource<https://ambit2.repdose-opentox.de:8443/ambit2/dataset/13> ;

ot:units "mmol" ;

= <http://www.opentox.org/Ontology.owl#LOEL> .

<https://ambit2.repdose-opentox.de:8443/ambit2/feature/56>

aot:Feature ;

dc:title "c_Animal" ;

ot:hasSource<https://ambit2.repdose-opentox.de:8443/ambit2/dataset/13> ;

ot:units "" ;

= <http://www.owl-ontologies.com/Ontology1288769658.owl#p1_species> .

<https://ambit2.repdose-opentox.de:8443/ambit2/feature/55>

aot:Feature ;

dc:title "k_Reference" ;

ot:hasSource<https://ambit2.repdose-opentox.de:8443/ambit2/dataset/13> ;

ot:units "" ;

= <http://www.owl-ontologies.com/Ontology1288769658.owl#p6_reference> .

<https://ambit2.repdose-opentox.de:8443/ambit2/feature/54>

aot:Feature ;

dc:title "h_Effect" ;

ot:hasSource<https://ambit2.repdose-opentox.de:8443/ambit2/dataset/13> ;

ot:units "" ;

= <http://www.owl-ontologies.com/Ontology1288769658.owl#p3_pathological_effects> .

ot:hasSource

aowl:ObjectProperty .

ot:units

aowl:DatatypeProperty .

<https://ambit2.repdose-opentox.de:8443/ambit2/feature/52>

aot:Feature , ot:NumericFeature ;

dc:title "e_Study NOEL im mmol" ;

ot:hasSource<https://ambit2.repdose-opentox.de:8443/ambit2/dataset/13> ;

ot:units "mmol" ;

= <http://www.opentox.org/Ontology.owl#NOEL> .

<https://ambit2.repdose-opentox.de:8443/ambit2/feature/53>

aot:Feature ;

dc:title "CAS" ;

ot:hasSource<https://ambit2.repdose-opentox.de:8443/ambit2/dataset/13> ;

ot:units "" ;

= ot:CASRN .

ot:NumericFeature

aowl:Class ;

rdfs:subClassOfot:Feature .
